# Dimension of chronic hepatitis C virus in HIV-infected patients in the interferon-free era: an overview from south Spain

**DOI:** 10.1007/s10096-015-2476-9

**Published:** 2015-09-05

**Authors:** A. Rivero-Juarez, A. Gutierrez-Valencia, M. Castaño, D. Merino, K. Neukam, M. J. Ríos-Villegas, M. A. Lopez-Ruz, P. Jiménez-Aguilar, M. Marquez, A. Collado, A. Gomez-Vidal, J. Hernandez-Quero, F. Tellez, E. Fernandez-Fuertes, A. Rivero, L. F. López-Cortés, on behalf of Grupo para el Estudio de las Hepatitis Víricas (HEPAVIR) de la Sociedad Andaluza de Enfermedades Infecciosas (SAEI)

**Affiliations:** Unidad Clínica de Enfermedades Infecciosas, Instituto Maimonides de Investigación Biomédica de Córdoba (IMIBIC), Universidad de Córdoba, Hospital Universitario Reina Sofía, Avda. Menendez Pidal s/n, 14004 Cordoba, Spain; Unidad Clínica de Enfermedades Infecciosas, Microbiología y Medicina Preventiva, Instituto de Biomedicina de Sevilla (IBiS), Hospital Universitario Virgen del Rocío/CSIC/Universidad de Sevilla, Seville, Spain; Unidad Clínica de Enfermedades Infecciosas, Hospital Regional Universitario Carlos Haya, Málaga, Spain; Unidad Clínica de Enfermedades Infecciosas, Complejo Hospitalario Universitario de Huelva, Huelva, Spain; Unidad Clínica de Enfermedades Infecciosas y Microbiología, Instituto de Biomedicina de Sevilla (IBiS), Hospital Universitario de Valme, Seville, Spain; Unidad Clínica de Enfermedades Infecciosas y Microbiología, Hospital Universitario Virgen Macarena, Seville, Spain; Unidad Clínica de Enfermedades Infecciosas y Microbiología, Hospital Universitario Virgen de las Nieves, Granada, Spain; Unidad Clínica de Enfermedades Infecciosas, Hospital Universitario Puerto Real, Cádiz, Spain; Unidad Clínica de Enfermedades Infecciosas y Microbiología, Hospital Regional Universitario Virgen de la Victoria, Málaga, Spain; Unidad Clínica de Enfermedades Infecciosas, Complejo Hospitalario Torrecárdenas, Almería, Spain; Unidad Clínica de Enfermedades Infecciosas, Complejo Hospitalario de Jaén, Jaén, Spain; Unidad Clínica de Enfermedades Infecciosas, Hospital Universitario San Cecilio, Granada, Spain; Unidad Clínica de Enfermedades Infecciosas y Microbiología, Hospital La Línea, AGS Campo de Gibraltad, Cádiz, Spain; Unidad de Medicina Tropical, Hospital de Poniente, Almería, Spain

## Abstract

The implementation of hepatitis C (HCV) direct-acting antiviral drugs is prioritized in several populations in which its application provides the most immediate and impactful benefit. In this scenario, a precise knowledge of the situation of human immunodeficiency virus (HIV)/HCV chronic co-infection is required to adequately address this disease. This cross-sectional study was performed in 21 hospitals in Andalusia (Spain). The study population consisted of HIV-infected patients with an active HCV chronic infection who were not receiving HCV treatment at the time of inclusion. A total of 13,506 HIV-infected patients were included in the study. Of them, 2561 (18.9 %) presented chronic HCV infection. The majority of the patients included were on highly active antiretroviral therapy (HAART; 96.2 %), showed plasma levels with an undetectable HIV viral load (92.5 %), and had a good immunological status (median CD4+ cell count of 486 cells/mL). The HCV genotype distribution was as follows: 58.1 % were genotype 1, 1.1 % were genotype 2, 16.1 % were genotype 3, and 22.1 % were genotype 4 (2.6 % were missing data). In total, 24.8 % of the patients showed liver fibrosis stage F0–F1, 27.9 % showed stage F2, 16.7 % showed stage F3, and 21 % showed stage F4 (9.6 % were missing data). With regards to previous HCV treatment experiences, 68.05 % of the patients were naïve and 31.95 % had failed to respond to a previous treatment. The burden of HCV/HIV co-infected patients in our population was reported as one in five HIV-infected patients requiring HCV treatment. The implementation of extra resources to face this important health challenge is mandatory.

## Introduction

The introduction of direct-acting antiviral (DAA) drugs has supposed a significant improvement in the prognosis of the hepatitis C virus (HCV) infection [1–6]. This important advancement has substantially increased the likelihood of achieving a sustained virological response (SVR) using shorter and safer therapies [1–6]. In consequence, the target population to be treated has expanded significantly because the lack of clinical contraindications, mainly due to interferon (IFN), is no longer a cornerstone of HCV treatment [7, 8].

It is estimated that ∼3 % of the world’s population (∼170 million people) is chronically infected with HCV, of which approximately 350,000 die annually from complications related to cirrhosis or hepatocellular carcinoma [9]. Among those particularly affected is the group of patients with concurrent human immunodeficiency virus (HIV) infection because both infections share identical routes of transmission [10]. The reduction of morbidity and mortality and the increased survival in patients with HIV infection, which is attributable to the effectiveness of antiretroviral therapy, has currently allowed HCV chronic liver disease to become a major cause of hospitalizations and mortality in these patients because survival is significantly lower, regardless of other prognostic markers of cirrhosis, compared to mono-infected patients [11–18]. Therefore, the HIV-infected population is a high-priority population that must receive immediate treatment against chronic HCV infection [7].

In this scenario, precise knowledge of the situation of the HIV/HCV chronic co-infection would facilitate the adequate provision of personal and material resources required to adequately address this disease in each center and in the community as a whole. Thus, the aim of our study was to evaluate the current situation of HCV chronic infection in HIV co-infected patients. Given these drawbacks, we designed a study with the objective of determining the dimension of the HCV chronic infection among HIV-infected patients.

## Methods

### Study design and source of information

**Fig. 1:**
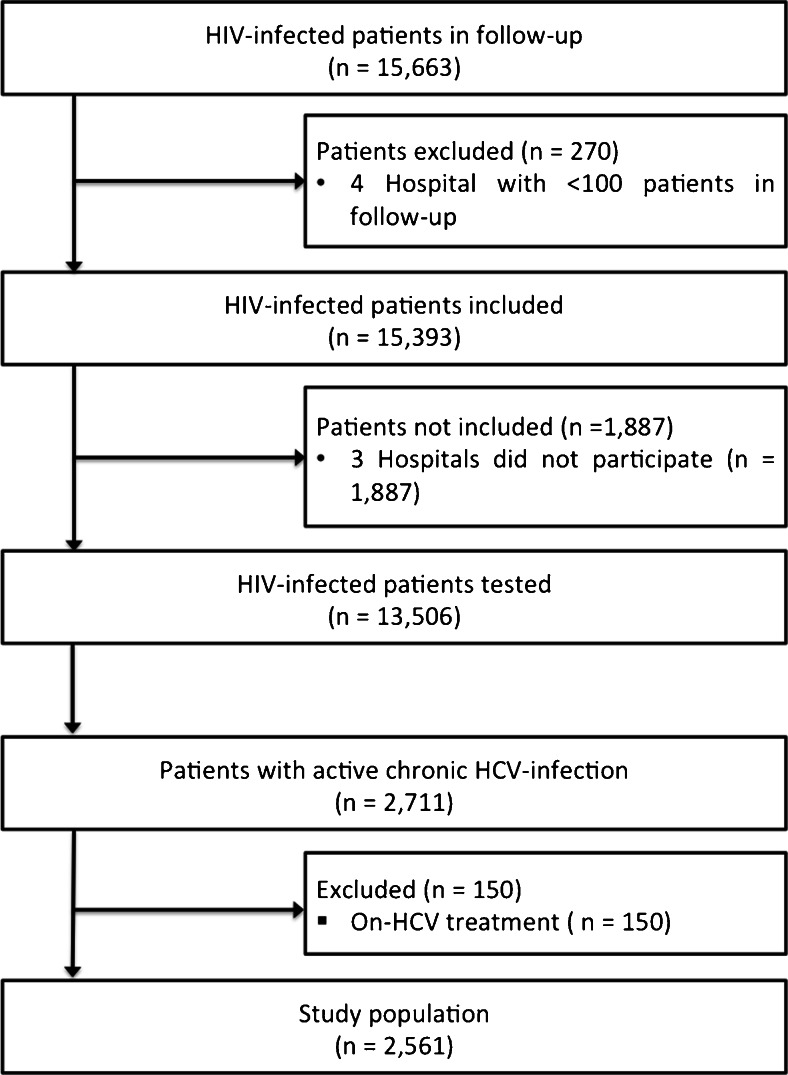
Patient flow diagram

This prospective cross-sectional study was conducted in two phases to address two specific objectives. During the first phase, an inquiry was performed in 21 hospitals that conformed to the HIV health care of the territory (Fig. 1), with the objective of calculating the total HIV-infected patient population. Once all the patients were obtained at the end of the first phase, the second phase selected hospitals with a significant number of patients in active follow-up, excluding those that attended <100 patients. The objective of the second phase was to identify the number of HIV-infected patients with active HCV chronic infection. To assess this objective, a specific electronic form (database) was facilitated to each hospital to record specific clinical data of each chronic HCV-infected patient (defined as the study population).

The study was designed, managed, and analyzed by all the authors who reviewed the study data, guaranteed the reliability of the data, conducted the study in accordance with the protocol followed, and approved the decision to submit the manuscript for publication.

### Setting

The study was performed in Andalusia, Spain (south Europe) during June and December 2014. Andalusia is a south Europe region with universal health coverage and a population of 8.3 million people. Andalusia features 21 hospitals encompassing the entire territory that attend HIV-infected patients.

### Participants

The study population consisted of HIV-infected patients with an active HCV chronic infection, which was identified during the second phase of the study. This population was defined as: (i) patients with detectable plasmatic HCV-RNA at the moment of inclusion and (ii) patients who were not on HCV therapy at the moment of inclusion in the study. Consequently, the data of patients with undetectable HCV viral loads, including those with SVR or with spontaneous viral clearance, were not collected in the study.

### Variable collection and definitions

The data were collected in electronic forms (database), including the participant, demographic, clinical, and virological characteristics. These data included age, gender, risk for HCV infection, current use of antiretroviral therapy (yes or no), current CD4+ cell count (cells/mL), current plasmatic HIV viral load (copies/mL), current plasmatic HCV viral load (IU/mL), HCV genotype/subtype, current liver fibrosis stage, Child–Pugh–Turcotte (Child-PT) score (A, B, or C), history of HCV therapy, and current use of HCV therapy.

An undetectable HIV viral load was defined as an HIV-1 RNA viral load of less than 50 copies per mL, using RT-PCR (Cobas TaqMan; Roche Diagnostic Systems Inc., Pleasanton, CA, USA). Liver fibrosis staging was performed by liver biopsy (following the METAVIR fibrosis score) and/or liver transient elastography (FibroScan; Echosens, Paris, France). The liver fibrosis stages were grouped as follows: (i) F0–F1 METAVIR fibrosis score or LSM <7.2 kPa; (ii) F2 METAVIR fibrosis score or LSM 7.2–8.9 kPa; (iii) F3 METAVIR fibrosis score or LSM 9–14.5; and (iv) F4 METAVIR fibrosis score or LSM ≥14.6 kPa. In the patients with liver cirrhosis, the Child-PT score was calculated using the parameters hepatic encephalopathy, ascites, total bilirubin, serum albumin, and prothrombin time. According to the value obtained, the patients were classified as follows: A (5–6 points), B (7–8 points), or C (>9 points).

The study data were transcribed onto data collection forms in which one code was assigned to each patient: the ID code. Only the physician directly responsible for each patient and/or authorized personnel had access to the medical history of the patients according to Law 15/1999 of December 13, Protection of Personal Data. This confidential information is the exclusive property of the physician directly responsible for patient care and may not be used except for conducting this study. The information created during the conduct of this clinical study is considered confidential and is/will be used by researchers in relation to the objectives of the study.

### Statistical methods

The elaboration of the manuscript has been performed following the Strengthening the Reporting of Observational Studies in Epidemiology (STROBE) recommendations. For the purpose of the study, those patients on HCV treatment at the moment of inquiry were excluded from the analysis. The descriptive statistics of the patients were reported. The continuous variables were summarized as the median and interquartile range (IQR); meanwhile, the categorical variables were expressed as frequencies/number of cases and percentages. The categorical variables were compared using the Chi-square test or Fisher’s exact test if the expected frequency of any group was lower than 5 %.

The principal analysis consisted of the patient distribution according to HCV genotype and liver fibrosis stage. A secondary analysis was performed in which the HCV genotype and liver fibrosis distribution were reported by sorting the patients according to previous experience with HCV treatment (including Peg-IFN/RBV and DAA-based regimens).

### Ethics issues

This study was conducted according to the principles of Good Clinical Practice (Ministry of Health, Royal Decree 223/2004 of 6 February) and the Declaration of Helsinki; the protection of personal data is guaranteed in accordance with Law 15/1999 of 13 December on the Protection of Personal Data and Royal Decree 1720/2007 of 21 December. The study did not require informed consent because the patients were not directly interviewed, and completely anonymous information from existing records was collected, ensuring the protection of personal data in accordance with Law 15/1999 of 13 December on Personal Data Protection.

The study coordinator presented the study protocol (protocol code: HEP-COI-1-2014) to the Coordinating Ethics Committee of Biomedical Research of Andalusia for evaluation and obtained approval (05/14). Those responsible for the providers of health services where the study occurred were given a copy of the protocol and the documents evidencing approval by the Ethics Committee in accordance with the procedures and legal requirements.

## Results

### Population tested

After the first phase, a total of 15,663 HIV-infected patients were obtained in the prospective follow-up in our media (HIV prevalence: 1.8 per 1000 inhabitants) (Fig. 1). For the second phase, four hospitals were excluded because they had less than 100 patients in their follow-up of an HIV-infected population (*n* = 270). Consequently, 15,393 HIV-infected patients (98.3 % of the total population in follow-up) comprised the target population of the study.

Fourteen of the 17 hospitals included in the second phase of the study agreed to participate (Fig. 1). Thus, the study target population consisted of 13,506 patients (87.4 % of the target population). Of this population, the presence of active chronic HCV infection was reported in 2711 patients (20.5 %), of which 150 patients (5.5 %) were on HCV treatment at inclusion and were excluded from the analysis according to the study protocol. Consequently, 2561 (18.9 %) HIV/HCV chronically infected patients constituted the study population.

### Patients

The baseline demographic and clinical characteristics are shown in Table 1. The majority of the patients included were on highly active antiretroviral therapy (HAART; 96.2 %), showed undetectable plasma HIV viral loads (92.5 %), and had a good immunological status (median CD4+ cell count: 486 cells/mL [308–697]). The most prevalent HCV genotype was genotype 1 (58.1 %), with HCV subtype 1a being predominant in our population (38.3 %). In total, 21 % of the patients (*n* = 538) showed liver cirrhosis, with a Child-PT score of A in 65.8 % of the patients in this subset.

**Table 1:** Baseline demographic and clinical characteristics of the study patients

Characteristic	Global (*n* = 2561)	G-1a (*n* = 572)	G-1b (*n* = 329)	G-2 (*n* = 28)	G-3 (*n* = 413)	G-4 (*n* = 565)
Age, years^a^	49 (45–52)	48 (44–53)	49 (45–52)	31 (26–57)	49 (45–52)	48 (45–52)
Age range, no. (%)						
<40	133 (5.3)	35 (6.1)	18 (5.4)	2 (7.1)	18 (4.3)	32 (5.6)
41–50	1352 (52.8)	304 (53.1)	149 (45.2)	11 (39.2)	207 (50.1)	317 (56.1)
51–60	1017 (39.7)	220 (38.4)	148 (44.9)	14 (50)	170 (41.1)	206 (36.4)
>60	59 (2.2)	13 (2.4)	14 (4.5)	1 (3.7)	17 (4.5)	10 (1.9)
Gender, no. (%)						
Male	2118 (82.7)	471 (82.3)	283 (86)	23 (82.1)	327 (79.1)	454 (80.3)
Female	443 (17.3)	101 (17.7)	46 (14)	5 (17.9)	86 (20.9)	111 (19.7)
Risk group for HCV infection, no. (%)						
IDU	2248 (87.7)	499 (87.2)	279 (84.8)	24 (85.7)	349 (84.5)	495 (87.6)
Heterosexual	219 (8.5)	42 (7.5)	32 (9.7)	3 (10.7)	49 (11.8)	54 (9.5)
Homosexual	75 (2.9)	21 (3.6)	14 (4.2)	1 (3.6)	12 (2.9)	12 (2.1)
Blood derived	19 (0.9)	9 (1.7)	3 (1.3)	0	1 (0.8)	2 (0.8)
HAART, no. (%)						
Receiving	2468 (96.2)	556 (97.2)	317 (96.3)	27 (96.4)	394 (95.3)	542 (95.9)
Non-receiving	93 (3.8)	16 (2.8)	12 (3.7)	1 (3.6)	19 (4.7)	23 (4.1)
HIV viral load, no. (%)^b^						
Undetectable	2370 (92.5)	527 (92.1)	301 (91.4)	27 (96.4)	383 (92.7)	521 (92.2)
Detectable	191 (7.5)	38 (7.9)	21 (8.6)	1 (3.6)	26 (7.3)	42 (7.8)
CD4+ total count, cells/mL^a^	486 (308–697)	470 (328–702)	502 (304–694)	400 (278–699)	452 (321–693)	513 (394–721)
CD4+ cell grading, no. (%)						
<200 cells/mL	339 (13.2)	83 (14.6)	31 (9.5)	1 (3.6)	62 (15.1)	64 (11.4)
≥200 cells/mL	2222 (86.8)	489 (85.4)	298 (90.5)	27 (96.4)	351 (84.9)	501 (88.6)
HCV genotype, no. (%)						
Genotype 1	1490 (58.1)	–	–	–	–	–
Genotype 2	28 (1.1)	–	–	–	–	–
Genotype 3	413 (16.1)	–	–	–	–	–
Genotype 4	565 (22.1)	–	–	–	–	–
Non-genotyped	65 (2.6)	–	–	–	–	–
HCV genotype 1 subtype, no. (%)						
Genotype 1a	572 (38.3)	–	–	–	–	–
Genotype 1b	329 (22.1)	–	–	–	–	–
Other genotype^c^	41 (2.7)	–	–	–	–	–
Non-subtyped	552 (36.9)	–	–	–	–	–
Liver fibrosis stage, no. (%)^d^						
F0–F1	636 (24.8)	147 (25.7)	79 (24)	8 (28.5)	89 (21.5)	145 (25.6)
F2	716 (27.9)	180 (31.4)	103 (31.3)	6 (21.4)	83 (20.1)	177 (31.3)
F3	428 (16.7)	81 (14.1)	52 (15.8)	5 (17.8)	81 (19.6)	95 (16.8)
F4	538 (21)	121 (21.1)	66 (20.1)	4 (14.2)	101 (24.4)	87 (15.5)
Not staged	243 (9.6)	43 (7.5)	29 (8.8)	5 (17.5)	59 (14.4)	61 (10.8)
Child-PT, no. (%)						
A	345 (65.8)	74 (61.5)	43 (65.1)	4 (100)	65 (64.3)	49 (56.3)
B	53 (9.8)	10 (8.2)	4 (6)	0	14 (13.8)	10 (11.4)
C	17 (3.1)	8 (6.6)	1 (1.5)	0	1 (1.2)	3 (2.5)
Not available	123 (21.3)	29 (23.7)	18 (27.4)	0	21 (20.7)	26 (29.8)
HCV therapy previous experience, no. (%)						
Naïve	1743 (68.05)	379 (66.2)	207 (62.9)	22 (78.5)	269 (65.1)	384 (67.9)
Non-responder to Peg-IFN/RBV	743 (29.01)	167 (29.1)	102 (31)	6 (21.5)	143 (34.6)	178 (31.5)
Non-responder to DAA-based regimen	75 (2.94)	26 (4.7)	20 (6.1)	0	1 (0.3)	3 (0.6)
Previous response to Peg-IFN/RBV, no. (%)						
Non-responders	451 (60.7)	93 (55.6)	53 (51.9)	6 (100)	35 (24.4)	130 (73)
Viral relapse	123 (16.5)	21 (12.5)	19 (18.6)	0	63 (44)	25 (14)
Therapy withdrawn^e^	169 (22.8)	53 (31.9)	30 (29.5)	0	45 (31.6)	23 (13)
Previous response to DAA-based regimen, no. (%)						
Non-responders	46 (61.3)	5 (19.2)	7 (37.1)	0	0	2 (66.6)
Viral relapse	13 (17.3)	17 (65.3)	11 (54.2)	0	1 (100)	0
Therapy withdrawn^e^	16 (21.4)	4 (15,5)	2 (18.7)	0	0	1 (33.4)
IFN-based therapy contraindication, no. (%)^f^	198 (7.7)	41 (7.1)	26 (7.9)	1 (3.5)	43 (10.4)	33 (5.8)
Alcohol abuse, no. (%)^g^	55 (2.1)	16 (2.7)	6 (1.8)	1 (3.5)	7 (1.6)	8 (1.4)

Number of cases (no.); percentage (%); genotype 1a (G-1a); genotype 1b (G-1b); genotype 2 (G-2); genotype 3 (G-3); genotype 4 (G-4); hepatitis C virus (HCV); injecting drug user (IDU); highly active antiretroviral therapy (HAART); human immunodeficiency virus (HIV); milliliter (mL); Child–Pugh–Turcotte score (Child-PT); pegylated interferon plus ribavirin (Peg-IFN/RBV); direct-acting antiviral (DAA) agent

^a^Expressed as the median (interquartile range)

^b^Undetectable plasmatic HIV viral loads were defined as HIV-RNA <50 IU/mL

^c^Genotypes 1c or 1a/b

^d^Liver fibrosis was measured by liver biopsy or liver stiffness

^e^Patients who voluntarily dropped out of therapy or withdrew because of adverse events

^f^Including patients with severe adverse events to previous interferon-based therapy, advanced or decompensated liver cirrhosis, oncological patients, those with severe renal impairment, uncontrolled psychiatric disorders, or autoimmune diseases

^g^Defined as a daily alcohol ingestion >100 mg

In regards to previous HCV treatment (Table 1), 68.05 % of the patients had not received previous therapy and, consequently, 31.95 % had failed to respond to at least one previous HCV treatment. Among the 818 patients with a previous treatment failure, 60.7 % of patients did not complete the full course of therapy because of the absence of a viral response, 16.6 % experienced a viral relapse after a full course of therapy, and 22.7 % voluntarily dropped out of therapy or withdrew because of adverse events.

### Distribution according to HCV genotype and liver fibrosis

**Fig. 2:**
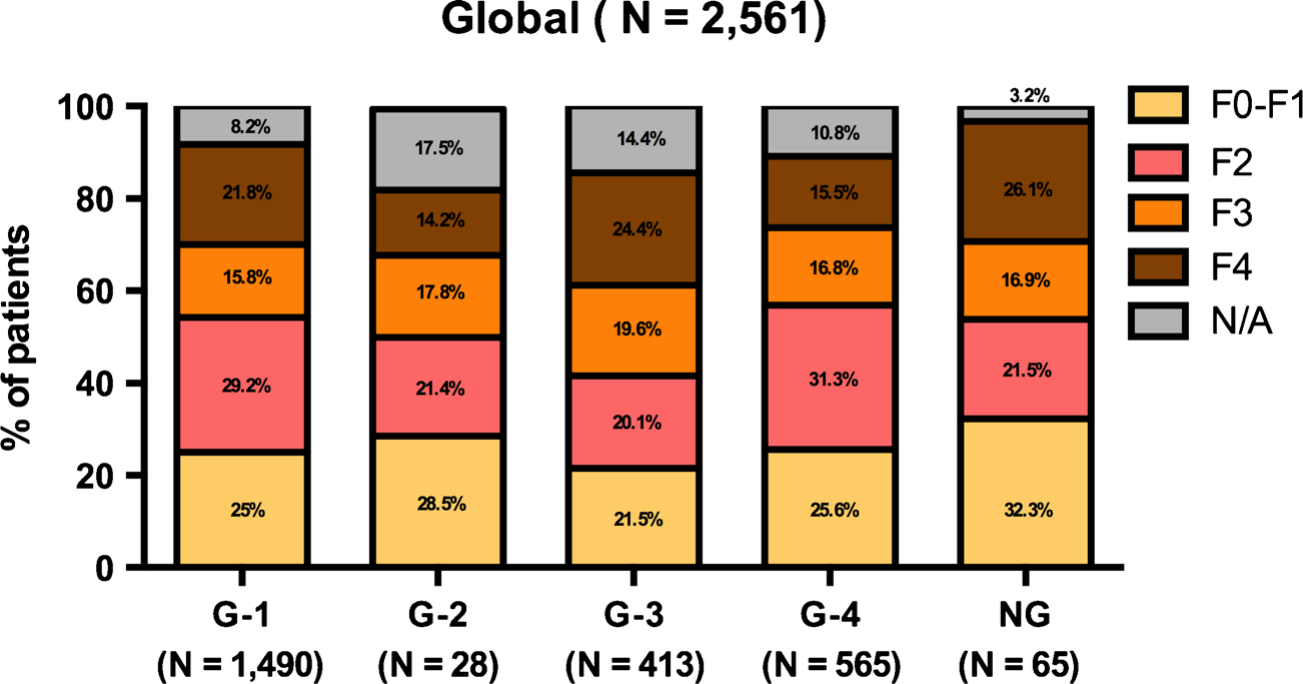
Liver fibrosis stage distribution according to hepatitis C virus (HCV) genotype in the total population included. The distribution is expressed as the cumulative percentage in each HCV genotype column. HCV genotype 1 (*G-1*), HCV genotype 2 (*G-2*), HCV genotype 3 (*G-3*), HCV genotype 4 (*G-4*), HCV genotype undetermined (*NG*), liver fibrosis stage undetermined (*N/A*)

The patient distribution according to HCV genotype and liver fibrosis stage is shown in Fig. 2. The liver fibrosis stage appeared to be consistent across HCV genotypes. The highest percentage of F4 patients was found among HCV genotype 3 patients (24.4 %). The highest percentage of F2 patients was found in those bearing HCV genotype 4 (31.3 %) but was not statistically significant. Among those patients with an unavailable HCV genotype (*n* = 65), 26.1 % were classified as F4 liver fibrosis stage. The percentage of missing liver fibrosis stages was greater than 10 % in the patients bearing HCV genotypes 2, 3, and 4.

### Distribution of HCV genotype and liver fibrosis according to previous HCV treatment

**Fig. 3:**
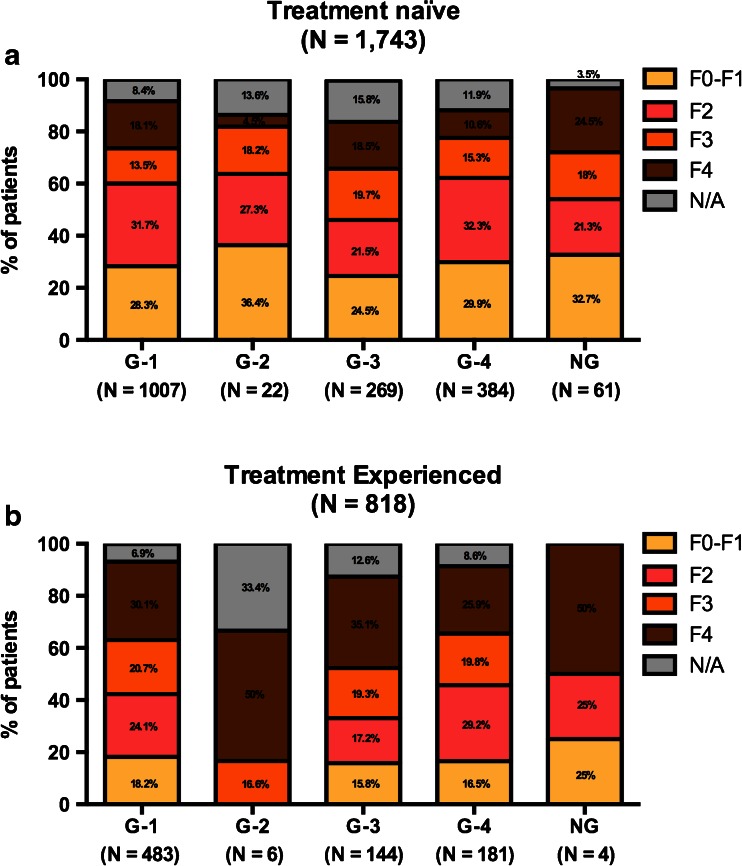
Liver fibrosis stage distribution according to HCV genotype in treatment-naïve (**a**) and treatment-experienced patients (**b**). The patients with previous treatment failure to pegylated-interferon plus ribavirin (Peg-IFN/RBV) or direct-acting antiviral (DAA) drugs therapy were grouped for the analysis. The distribution is expressed as the cumulative percentage in each HCV genotype column. HCV genotype 1 (*G-1*), HCV genotype 2 (*G-2*), HCV genotype 3 (*G-3*), HCV genotype 4 (*G-4*), HCV genotype undetermined (*NG*), liver fibrosis stage undetermined (*N/A*)

The secondary analysis was performed to assess the HCV genotype and liver fibrosis stage distribution according to previous HCV therapy (Fig. 3). This distribution among previously untreated and treatment-experienced patients is shown in Fig. 3a, b, respectively. Globally, the percentage of F4 patients was the greatest among treatment-experienced patients than previously untreated patients (30.4 % vs. 16.6 %, *p* < 0.001). In contrast, the percentage of F0–F1 patients was higher among treatment-naïve patients (28.3 %) than in treatment-experienced patients (16.9 %) (*p* < 0.001). When this association was analyzed in each treatment experience group according to HCV genotype, no statistical association was found.

## Discussion

This is the first study showing the dimensions of HCV chronic infection in HIV-infected patients by targeting one of the largest HIV population samples. The burden of HCV/HIV co-infected patients in our population reported that at least one in five HIV-infected patients required HCV treatment. Therefore, the implementation of extra resources to face this important health challenge is mandatory.

In Europe, the most common HCV genotype is genotype 1 (specifically, genotype 1b), followed by genotype 3; there is a relatively low frequency of genotypes 2 and 4 in the overall HCV population [19]. Nevertheless, if the HCV genotype distribution is focused among HIV co-infected patients, this appears to change. HCV genotypes appear to have strong epidemiological behavior; in other words, they have a close relationship with HIV infection/transmission [19, 20]. The main route of HCV transmission in HIV-infected patients has been illegal injection drug use (IDU) [21]; in our study, this route represented 87.7 % of the transmission. In this context, the introduction of several HCV genotypes into networks of IDU in Europe, such as genotypes 1a and 4 [19], has altered the HCV genotype distribution in this population subset. This point was confirmed in our study, in which HCV genotype 1 (specifically, genotype 1a) and HCV genotype 4 were the most prevalent, as opposed to HCV genotype 3. This particular HCV genotype distribution converted these patients to “difficult to treat patients”, mainly because of the significantly low response rate to Peg-IFN/RBV therapy compared to HCV genotypes 2 and 3 [22].

IDU HCV-transmitted patients, specifically those co-infected with HIV, show a faster liver fibrosis progression to liver cirrhosis and end-stage liver disease [23, 24]. Mainly because of this reason, historically, HIV-infected patients have constituted the worst subset of HCV-infected patients. However, this axiom was established during the era in which HCV therapeutic management was insufficient in terms of efficacy and safety. Taking this into account and considering the availability of more effective antiviral drugs against HCV and HIV infection, this situation may have changed. In our study, the majority of patients were on HAART (96.2 %), showed undetectable HIV viral loads (92.5 %), and had a relatively good immunological situation (406 CD4+ cells/mL median value). This population (likely comparable to the population followed in other countries with universal health care coverage) matched the population included in clinical trials in which the safety and efficacy of HCV DAA drugs were tested in HIV-infected patients. In these trials, the SVR rates obtained were similar to those obtained in non-HIV co-infected patients, across liver fibrosis stages, genotype, or previous treatment experience [25–27]. Therefore, HIV infection should not remain an associated risk factor to achieve a successful HCV treatment outcome in the new DAA era and should benefit from the same treatment regimen and strategies as HCV mono-infected patients. On the other hand, more than 40 % of our population was aged more than 50 years. This establishes a high sensitivity population due to polymedication (including HAART drugs) and age-associated pathologies, supposing a higher drug–drug interaction complicating the management of this population using HCV DAA drugs.

The HCV clinical guidelines identify those HCV-infected patients at the highest risk for severe hepatic complications [7, 8]. The main reason is that this population includes patients in whom HCV therapy provides the most immediate and impactful benefit [28, 29]. Our study shows that an important burden of the highest priority patients require an immediate treatment implementation to improve life expectancy and avoid both liver and non-liver-related complications. According to liver fibrosis stage, in our study, the proportion of patients with a liver fibrosis stage of F3 or F4 increased to 37.7 %, with the highest representation of patients who had failed to respond to a previous treatment. However, the different distribution of the F4 population according to previous HCV treatment experience was potentially a result of the prioritization of HCV treatment when non-highly successful drugs were available. HCV genotype 3 has been considered one of the best scenarios under the Peg-IFN/RBV therapy. Nevertheless, those patients who have currently failed to respond to a previous treatment and with liver cirrhosis staging (35.1 % in our population) do not have a good prognosis [30]. This is because, by applying the treatment regimens currently recommended in this population, the expected SVR rate would be approximately 60 %, which is clearly insufficient compared to the SVR rate achieved in the same population in other genotypes. Therefore, this represents a priority treatment population with an expected lower healing rate. Consequently, specific treatment strategies must be investigated in this population.

Our study has several limitations. Firstly, it should be noted that 9.6 % of patients had missing liver fibrosis stage data. Secondly, although the percentage of ungenotyped patients was relatively low (2.6 %), the percentage of HCV genotype 1 patients who were unsubtyped was 36.9 %. Nevertheless, the missing data were distributed across all hospitals; thus, the reported liver fibrosis stage and HCV genotype distribution could not change significantly. This point supports the fact that the implementation of extra resources is mandatory to obtain the best management and care for HCV infection.

In conclusion, our results reveal the current magnitude of this disease, showing that, during the next few years, HCV treatment should be a mandatory clinical provision in HIV-infected patients. In this situation, acquiring additional resources to proportionate the best clinical care options in this highly sensitive population is a priority. In a public health care system, such as those in Spanish and most European countries, this supposes an important amount of resources to intensify care management in this area.
